# Neural Tube Defects and Folate Deficiency: Is DNA Repair Defective?

**DOI:** 10.3390/ijms24032220

**Published:** 2023-01-22

**Authors:** Xiuwei Wang, Jialu Yu, Jianhua Wang

**Affiliations:** Beijing Municipal Key Laboratory of Child Development and Nutriomics, Translational Medicine Laboratory, Capital Institute of Pediatrics, Beijing 100020, China

**Keywords:** neural tube defects, DNA damage, DNA repair, folate

## Abstract

Neural tube defects (NTDs) are complex congenital malformations resulting from failure of neural tube closure during embryogenesis, which is affected by the interaction of genetic and environmental factors. It is well known that folate deficiency increases the incidence of NTDs; however, the underlying mechanism remains unclear. Folate deficiency not only causes DNA hypomethylation, but also blocks the synthesis of 2′-deoxythymidine-5′–monophosphate (dTMP) and increases uracil misincorporation, resulting in genomic instabilities such as base mismatch, DNA breakage, and even chromosome aberration. DNA repair pathways are essential for ensuring normal DNA synthesis, genomic stability and integrity during embryonic neural development. Genomic instability or lack of DNA repair has been implicated in risk of development of NTDs. Here, we reviewed the relationship between folate deficiency, DNA repair pathways and NTDs so as to reveal the role and significance of DNA repair system in the pathogenesis of NTDs and better understand the pathogenesis of NTDs.

## 1. Introduction

Neural tube defects (NTDs) are common complex birth defects of the central nervous system that result from failure of neural tube closure during embryogenesis, including anencephaly, encephalocele and spina bifida, et al. [1,2]. NTDs are the second most common birth defects in humans [3,4], with an incidence of 0.5 to 5 per 1000 births [4,5,6,7]. The etiology of NTDs involves genetic and environmental factors, such as folate deficiency, which is an important cause of NTDs. Data collected over the last 40 years have demonstrated that the periconceptional use of folate reduced the population burden of NTDs, and more than 240 genes have been identified to be involved in neural tube closure [8]. Studies have demonstrated that while a large number of factors including genetic, nutritional, and environmental factors are related to the occurrence of NTDs, the underlying mechanism is not well known, which brings great difficulties to the targeted prevention and treatment of NTDs.

DNA repair is a series of biochemical reactions and molecular events that is related to restoring normal DNA sequence structure and maintaining the relative stability of genetic information, such as endogenous damage (including errors in DNA replication and spontaneous chemical changes) and exogenous damage (X-rays, ultraviolet (UV) radiation, and chemicals) in cells. The main biological effects of DNA damage are gene mutation and cell apoptosis. Studies have shown that DNA damage repair may contribute to the pathogenesis of NTDs [9,10,11,12]. Chemotherapeutic agents such as cyclophosphamide and methotrexate induce DNA damage and cell apoptosis, which is likely to be one of the mechanisms in the occurrence of NTDs [13,14,15,16,17,18,19]. Therefore, DNA damage repair pathways may function as a direct factor affecting the normal development of neural tubes at the molecular and cellular levels. This article reviews the research progress on the pathogenesis of NTDs, especially the association between folate deficiency and DNA repair.

## 2. Neural Tube Development and NTDs

**Figure 1:**
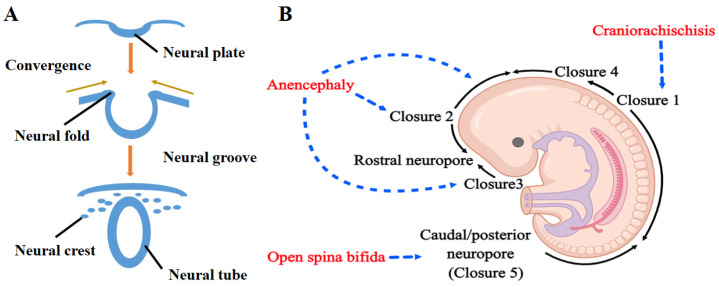
The schematic diagram of neural tube formation and the development of NTDs in human. (**A**) Diagram of neural tube formation. Neural plate bends to create the neural folds, which elevate towards the dorsal midline, and finally fuse to complete the formation of neural tube; (**B**) diagram of neural tube closure and the development of NTDs in human embryos (created with https://image.medpeer.cn, accessed on 9 January 2023). Neural tube closure is a discontinuous process initiated at closure 1, which may occur in the mid-cervical region. Failure of closure 1 results in craniorachischisis. Closure also initiates at Closure 3 (the rostral extremity of the forebrain). Fusion from the initial sites spreads bi-directionally into the cranial region and along the spinal region, finally completed by closure of the anterior and posterior neuropores (shown by black arrows). Failure of Closure 2 or Closure 3 results in anencephaly. Open spina bifida results from failure of posterior neuropore (Closure 5) closure.

The central nervous system, including the brain and spinal cord in vertebrates, arises initially from the neural tube, which is essential for the growth and development of the embryo. The neural tube is formed from the neural plate, which is specified and differentiated from the dorsal surface ectoderm after gastrulation proceeds. Then, the neural plate bends to create the neural folds, elevates towards the dorsal midline, and fuses to form the neural tube [20] (Figure 1A). Neural tube closure depends upon several biological processes such as convergent extension of the neural plate, neural crest cell migration, and neuroepithelial apoptosis, proliferation and differentiation. Impairment of the above-mentioned processes can affect proper neural tube development and closure, resulting in the occurrence of NTDs.

**Figure 2:**
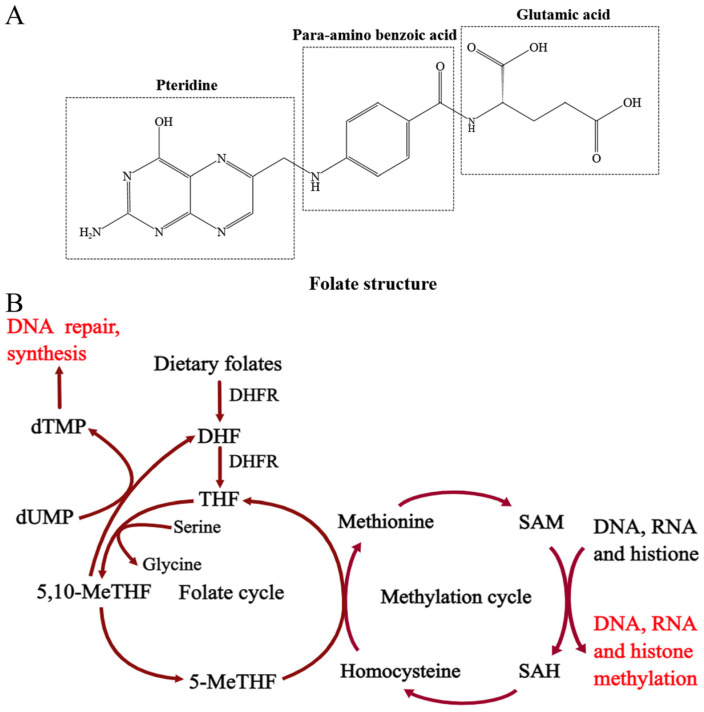
Folate structure and its metabolic pathway. (**A**) folate structure; (**B**) folate metabolic pathway. DHF: dihydrofolate; THF: tetrahydrofolate; DHFR: dihydrofolate reductase; 5-MeTHF: 5-Methyltetrahydrofolate; 5,10-MeTHF: 5,10-methylenetetrahydrofolate; dUMP: 2′-deoxyuridine-5′-monophosphate; dTMP: 2′-deoxythymidine-5′–monophosphate; SAM: S-adenosylmethionine; SAH: S-adenosylhomocysteine.

Neural tube closure is a discontinuous process. In humans, there are probably five closure sites of the neural tube (Figure 1B). Closure 1 is located at the junction of the spinal cord and hindbrain (mid-cervical region) and this closure spreads bidirectionally [21,22,23]. Closure 2 is located at the midbrain and forebrain boundary with closure also spreading bidirectionally [21,22,24]. Copp et al. found that Closure 2 did not appear to occur in human embryos [24]. Closure 3 is located at the rostral extremity of the neural plate with closure spreading caudally from this site [21,22,23]. Closure 4 is located at the caudal end of the rhombencephalon and proceeds rostrally to meet the caudal Closure 2 [21,22,23]. Closure 5 is located at the most caudal end of the neural tube and proceeds rostrally to meet the caudal Closure 1 [22]. Failure of neural tube closure at different regions leads to different abnormalities during early embryonic development in vertebrate embryos. Failure at the anterior section (Closure 2 or Closure 3) of the embryo results in exencephaly/anencephaly [24], and failure at the caudal portions (Closure 5) of the embryo result in spina bifida [4,25,26,27]. If the neural tube from the midbrain to the low spine (Closure 1) fails to fuse, craniorachischisis occurs [28] (Figure 2B). The etiology of NTDs is multifaceted. A large number of candidate genes and environmental factors were identified via epidemiologic studies and experimental models [29,30]. There are still challenges to confirm the primary etiology of NTDs for individual patients. Among environmental factors, folate deficiency is closely related to the occurrence of NTDs. Much evidence has confirmed that folate supplementation during the periconception period could reduce the risk of NTDs in offspring [31,32,33]. However, the underlying mechanism of folate-deficiency-induced NTDs remains unclear.

## 3. Folate Metabolism and NTDs

**Figure 3:**
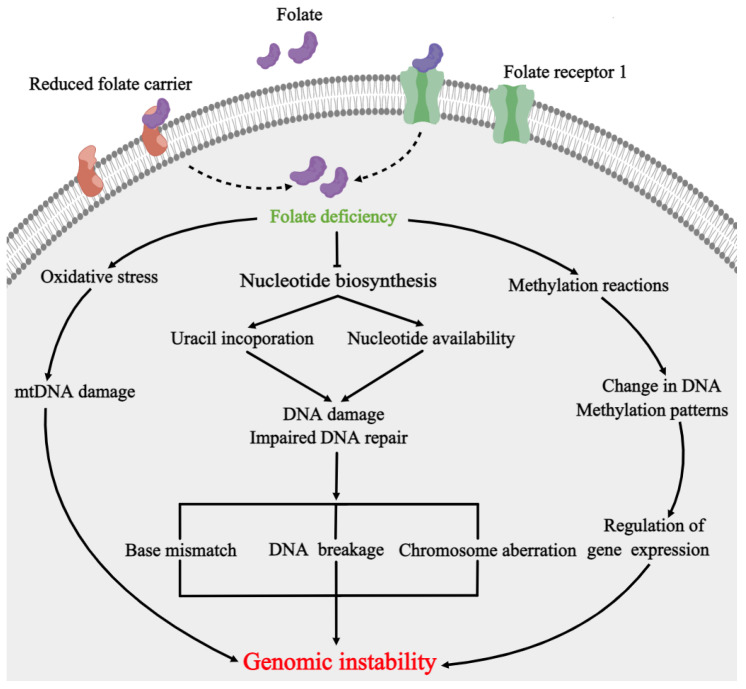
The association of folate deficiency and genomic instability. Folate is taken into cells through Folate receptor 1 or Reduced folate carrier to be used in nucleotide biosynthesis, methylation reaction and oxidative stress. Folate deficiency affects the normal biological functions of the cell, resulting in the impairment of genomic stability. Folate deficiency induces increase in uracil incorporation and decrease in nucleotide availability, which causing DNA damage that cannot be repaired efficiently, leading to base mismatch, DNA breakage, and chromosome aberration. As methyl donors, low folate can alter methylation patterns, resulting in changes in gene expression. Folate deficiency also causes oxidative stress with consequences that affect the integrity of mitochondrial DNA.

Folate, a water-soluble Vitamin B, is an essential micronutrient for cell survival in mammals. Folate is provided in the diet, especially from fruits and green leafy vegetables. It cannot be synthesized in vivo and must be obtained exogenously. Folate, which occurs predominantly as polyglutamate forms in the diet, consists of three parts: pteridine, para-aminobenzoate, and a glutamyl chain (Figure 2A). The polyglutamates are hydrolyzed to monoglutamate form for absorption on the brush border of enterocytes in the proximal small intestine [34]. Intracellular folate itself has no cofactor activity until it is converted into tetrahydrofolate (THF) by dihydrofolate reductase (DHFR). THF, as a biologically active form of folate, plays a critical role in one-carbon metabolism. THF functions as a one-carbon carrier, obtaining the one carbon from serine to generate glycine and 5,10-methylene-THF, which acts as a cofactor, providing methyl groups for the conversion of 2′-deoxyuridine-5′-monophosphate (dUMP) into dTMP; this is crucial for DNA synthesis. Furthermore, 5,10-methylene-THF is converted to 5-Methyl-THF, which acts as a methyl donor for homocysteine remethylation and cellular methylation reactions via its conversion to S-adenosylmethonine (SAM) (Figure 2B). Thus, low folate status not only causes dUMP accumulation and impairs the de novo dTMP synthesis pathway [35], but also results in the impairment of the methylation pattern by reducing the methyl donor [36], and causes oxidative stress with consequences affecting the integrity of mitochondrial DNA [37], leading to genomic instability (Figure 3).

Folate deficiency has been shown to be closely related to the occurrence of NTDs. Zhang et al. found that serum concentrations of folate from pregnant women with NTDs (geometric mean 9.55 nmol/L, P5-P95, 4.17–19.11 nmol/L) were significantly lower than those in control pregnant women (geometric mean 12.05 nmol/L, P5-P95, 5.41–38.77 nmol/L), and folate intake insufficiency (below 7.01 nmol/L in serum) increased the risk of NTDs [38]. Periconceptional supplementation of folate (0.4 mg/day) reduced the incidence of NTDs [39,40]. A China-U.S. collaborative project found that a 79% reduction in areas with high rates of NTDs (6.5 per 1000) and a 41% reduction in areas with low rates of NTDs (0.8 per 1000) was observed following 0.4 mg/day folate supplements during the peri-conception period in China [40]. Pathological studies found that folate-deficiency-induced abnormal DNA methylation involved in the occurrence of NTDs [41,42,43]. The methylation status of long interspersed nucleotide element-1 (LINE-1) is a good predictor of genome-wide methylation on the basis of cellular 5-methylcytosine. Wang et al. observed a significantly lower methylation level of LINE-1 in the nervous tissues of fetuses with NTDs compared to the control samples (54.57% vs. 59.03%), along with lower maternal plasma folate concentrations. The methylation levels of all CpG sites within LINE-1 in cephalic malformations (NTDs subgroup) were significantly lower than in the controls [41]. Increased methylation levels of the germline differentially methylated region (gDMR) in the paternally expressed gene 10 (PEG10)/sarcoglycan epsilon (SGCE) imprinted cluster with low folate levels (0.06 ± 0.01 ng/mg) were observed in spina bifida fetus brain tissues compared to control samples (folate levels, 0.14 ± 0.01 ng/mg). The high methylation level increased the risk of spina bifida (*OR* = 9.93, 95% *CI*, 1.86–53.07). Studies using HCT-15 cells suggest that folate-deficiency-mediated dysregulation of gDMR methylation of the *PEG10/SGCE* cluster is involved in spina bifida [43]. It has been reported that the fibroblast growth factor (FGF) pathway is disturbed in human encephalocele, with folate deficiency in NTD-affected fetal brain (0.06 ± 0.03 ng/mg), compared to control fetal brain (0.20 ± 0.05 ng/mg). This observation was also demonstrated in folate-deficient cells and mice model, indicating that folate deficiency during early embryonic development leads to disturbance of the FGF pathway and resulted in NTDs [44]. Meanwhile, the demethylation of H3K79 (histone H3 lysine 79) was inhibited under low folate conditions, which is associated with the development of NTDs [45]. These studies indicate that methylation change caused by folate deficiency is closely related to NTDs.

In addition to influencing the methylation pattern during early embryonic development, folate deficiency also causes the occurrence of NTDs by influencing DNA synthesis. It has been reported that folate deficiency impairs folate-dependent thymidylate biosynthesis, which results in excessive uracil incorporation into DNA, leading to genomic instability, such as single- and double-strand DNA breaks, chromosome breakage, and micronucleus formation [46,47,48]. These data indicate that folate deficiency could affect the ability of DNA repair and genomic stability [49,50]. Uracil misincorporation into DNA under folate deficiency might be causal in the development of folate-responsive NTDs [9,35]. Anti-cancer drugs such as antifolates (5-fluorouracil, methotrexate, and raltitrexed) reduced the de novo dTMP synthesis and increased the incorporation of uracil into DNA, resulting in the impairment of DNA synthesis [51,52,53]. Excessive uracil incorporation into DNA promoted cell apoptosis [35]. Treatment with 5-fluorouracil, methotrexate or raltitrexed promoted cell apoptosis and increased the occurrence of NTDs in mice [14,16,18,54]. Evidence from a genome-wide copy number variants (CNVs) study suggests that CNVs in ciliogenesis pathways are associated with NTDs [55]. These studies indicate that folate-deficiency-induced genomic instability might be one of the pathogenetic mechanisms of NTDs.

## 4. DNA Repair Pathways Involved in Folate Deficiency and NTDs

**Figure 4:**
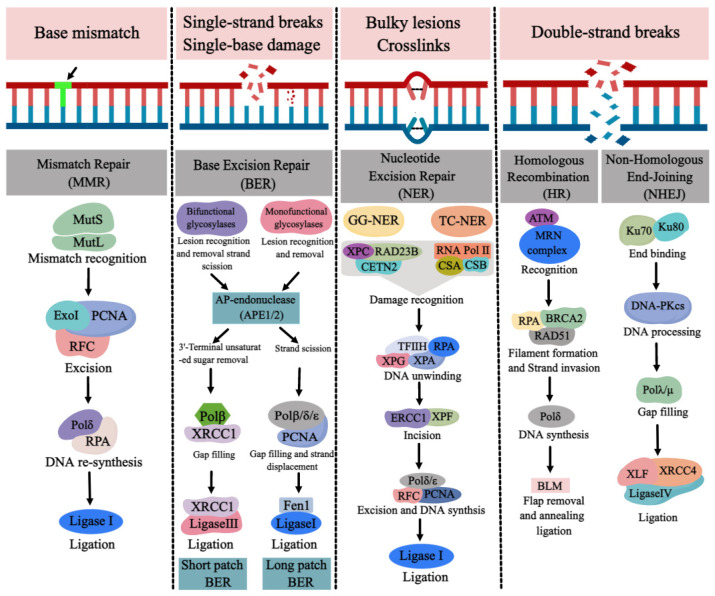
The major types of DNA repair pathways. Base mismatch is repaired by mismatch repair (MMR), single-strand breaks (SSBs) and single-base damage is repaired by base excision repair (BER). Bulky lesions and crosslinks are repaired by nucleotide excision repair (NER) and double-strand breaks (DSBs) are repaired by homologous recombination (HR) and nonhomologous end joining (NHEJ). (Created with https://image.medpeer.cn, accessed on 30 November 2022).

DNA repair pathways are essential for ensuring normal DNA synthesis, genomic stability, and integrity, which are required for a multitude of cellular processes such as cell proliferation, differentiation, cell cycle, apoptosis, and the development of tissues and organs. Defects or inappropriateness in DNA repair pathways are associated with detrimental health effects, including birth defects, cancer, and neurodegenerative diseases [56,57,58,59]. DNA repair genes are over-expressed at the early stages of normal embryonic development in order to reduce possible replication errors and genotoxic damage [60]. About 150 human DNA repair genes have been identified, involving the following DNA repair pathways: mismatch repair (MMR), base excision repair (BER), nucleotide excision repair (NER), and double-strand break repair (DSBR) [61,62] (Figure 4). Furthermore, DNA repair genes such as *p53* and *BRCA1/2* are crucial for embryogenesis and central nervous system development [63,64,65,66]. Loss of *p53* (*p53*^−/−^) in mouse embryos was demonstrated to cause a portion of exencephaly as well as spina bifida [67], while mouse embryos deficient in *BRCA1* also demonstrated varying degrees of anencephaly and spina bifida [68]. O6 methylguanine DNA methyltransferase (MGMT) is an efficient direct repair enzyme for DNA damage that can repair O6 methylguanine damage in a DNA sequence. Tran et al. reported that increased *MGMT* gene expression and *MGMT* promoter methylation contributed to the etiology of NTDs (especially in the female fetus) [69]. Therefore, the occurrence of NTDs may be related to the abnormality or deletion of various DNA repair pathways. Due to the link between folate deficiency and NER not being particularly evident, we mainly review the progress of DNA repair pathways (BER, MMR, DSBR) and NTDs with folate deficiency.

### 4.1. MMR and NTDs

The MMR pathway, including mutL homolog 1 (MLH1), MutS protein homologue 2 (MSH2), MutS homologue 6 (MSH6) and PMS1 homologue 2 (PMS2), is responsible for the surveillance and correction of errors during DNA replication, repair, and recombination. MSH2 couples with MSH6 or MSH3, forming MutSα or MutSβ complexes, respectively, and MLH1 couples with PMS2, PMS1 or MLH3, forming MutLα, MutLβ or MutLγ complexes, respectively [70]. MutS and MutL are ultimately responsible for the recognition of mismatches and insertion–deletion loops, after which the MLH1/PMS2 complex is recruited to degrade the mutated stretch and initiate resynthesis [71,72] (Figure 4).

Microsatellite instability (MSI) can reflect the instability of the genome, which is caused by inactivating a series of DNA MMR genes, especially *MLH1* [73]. Moreover, MSI is related to hypermethylation of the *MLH1* promoter region (which can lead to gene inactivation) in tumors; thus, MSI can reflect the deletion or dysfunction of the MMR system [74]. Studies have shown that folate has a role in regulation of the MSI. A study of folate levels and MSI statuses in the colonic mucosa of 26 patients with chronic ulcerative colitis found that MSI was found in 3/23 patients (13%) with ulcerative colitis. The three patients with MSI exhibited lower levels (30–50%) of folate concentrations in their serum, whole blood and colonic mucosa. Interestingly, after one of the patients with MSI received a folate supplement (5 mg/day) for 6 months, three of six microsatellite markers became stable, suggesting a close relationship between folate status and MSI [75]. Liu et al. studied the genomic instability in human fetuses with NTDs under folate deficiency; the results demonstrated that the total positive rate of MSI in the NTDs group was 46% (23 of 50 NTD patients) and *hMLH1* promoter demonstrated two specific methylation patterns at “4,5 CpG” sites in the target region, which were a 180-bp fragment upstream from exon-1 of *hMLH1* (−271 to −92 bp, containing 11 CpG sites) in some NTD samples [76]. These results suggest that folate-deficiency-induced occurrence of NTDs were associated with the abnormal function of the MMR system. It has been reported that H3K36me3 (histone H3 trimethylated lysine 36) recruits MutSα (MSH2-MSH6) onto chromatin through direct interactions with the MSH6 PWWP domain to regulate the MMR in vitro [77]. *Hypb* encodes the H3K36me3 specific methytransferase, which is closely related to NTDs, as observed by knocking it out in mouse embryos [78]. The targeted sequencing analysis of human NTDs with folate insufficiency found that a great majority of variants found were single-nucleotide substitutions or base–base mismatches that were repaired by MMR, and the most potentially deleterious variants were observed within H3K36me3 occupancy regions (68.1% of total occurrences). The co-immunoprecipitation and MSI analysis found that low folate status attenuated the binding of Msh6 with H3K36me3 and resulted in impairment of the MMR pathway, suggesting that folate-deficiency-induced NTDs in humans could be mediated via an adverse impact on MMR [79]. The methy-CpG binding domain protein 4 (MBD4) recognized T/U or T/G mismatch in the methylated CpG nucleotide sequence, which appeared to coordinate MMR with DNA methylation events. The C-terminal domain of MBD4 interacted with MLH1, which was involved in MMR [80,81,82]. It has been reported that *Mbd4* gene and *Ift122* gene share the same starting sequence (Exon 1) at the murine locus, and *Mbd4* exon 1–3 mutation abrogates the expression of both *Mbd4* and *Ift122*. Mouse embryos defective in *Ift22* gene (Exon 1–3) ranging from E10.5 to E13.5 demonstrated a complex phenotype characterized primarily by failure of neural tube closure (such as exencephaly) [10]. It was speculated that the *Mbd4* gene might interact with the *Ift122* gene at the transcriptional level and participate in the formation of mouse embryonic malformation. Therefore, the alteration of epigenetic modification (methylation of DNA or histone) induced by folate deficiency or mutation in MMR genes contributes to the closure of the neural tube.

### 4.2. BER and NTDs

The BER pathway is a highly conserved process to repair the oxidative damage, alkylation, deamination and methylation of DNA bases in cells [83,84]. DNA glycosylases in BER pathway recognize and remove base damage in three types: monofunctional, bifunctional, and nei-like DNA glycosylases [85]. Monofunctional enzymes such as uracil DNA glycosylase (UNG), single-strand selective monofunctional uracil DNA glycosylase (SMUG1), G/T mismatch-specific thymine DNA glycosylase (TDG), MBD4, N-methylpurine DNA glycosylase (MPG), and adenine DNA glycosylase (MUTYH) excise the damaged base, leaving an apurinic/apyrimidinic (AP) site and a phosphodiester backbone, which is further hydrolyzed by AP endonuclease1 (APE1), forming a single-strand break (SSB) with 5′-deoxyribosephosphate and 3′-hydroxyl ends [85,86]. Bifunctional glycosylases, including endonuclease-like protein 1 (NTH1) and 8-oxoguanine DNA glycosylase 1 (OGG1), remove the base and cleave the phosphodiester bond, leaving a β-elimination, which is hydrolyzed by APE1 and creates a nucleotide gap. Nei-like DNA glycosylases (NEIL1, NEIL2, NEIL3) catalyze a β/δ-elimination reaction, where the phosphodiester bond is cleaved, and a 3′-phosphate group is generated by polynucleotide kinase phosphatase (PNKP) [87,88]. The poly (ADP-ribose) polymerase 1(PARP1), as a DNA damage sensor, protects the strand break and recruits the DNA repair protein by its poly (ADP-ribosyl)ation activity [89]. DNA polymerase β (Polβ) in BER pathway fills the gap and removes the 5′-deoxyribosephosphate [90]. Finally, DNA ligase IIIα (LigIIIα) interacts with X-ray repair cross-complementing protein 1 (XRCC1) to complete the short-patch BER, which is responsible for repairing the single nucleotide gap [91,92]. Long-patch BER is employed to repair the gap of 2–10 nucleotides by proliferating cell nuclear antigen (PCNA)-dependent DNA polymerases δ/ε (Polδ/ε) to displace the damaged strand. Flap endonuclease 1 (FEN1) removes the displaced strand, leaving a nick ligated by the DNA ligase I (LigI) [93,94] (Figure 4).

The BER pathway is necessary for normal brain development and is closely related to neurodevelopmental disorder [95]. Folate deficiency results in DNA damage accumulation due to incapacitation of the BER [96,97,98]. The effect of folate deficiency on BER shows that the balance and coordination of BER in mice are impaired by increasing the UDG protein level (30%) without subsequently stimulating the APE1 and Polβ, resulting in a greater accumulation of DNA damage in response to folate deficiency [96]. Folate deficiency impacts BER capacity by inhibiting Polβ expression at the transcription level through the negative regulatory factors binding to the folate response region within the core promoter of Pol β in mice [97]. These studies indicate that folate deficiency decreases the capacity of BER by affecting the expression of the key enzymes in BER, which might contribute to the occurrence of NTDs. Olshan et al. conducted a case–control analysis from 250 NTDs (including 125 spina bifida, 125 oral clefts) and 350 non-malformation controls to investigate the relationship between DNA repair gene polymorphisms and NTDs; the results show that the polymorphism of BER gene *APE1* (Asp148Glu) reduced the risk of spina bifida, while, on the other hand, the BER genes *XRCC1* (Arg399Gln) and *OGG1* (Ser326Cys) could increase the risk of spina bifida [10]. Our previous study found that the polymorphisms (rs3136817, rs77794916, and rs1760944) of BER gene *APE1* in fetal brain tissues from 165 NTD fetuses and 300 control fetuses were statistically associated with NTDs under low folate status for Han population in a high-risk area of China. The allele C of rs3136817, allele T of rs77794916, and allele G of rs1760944 were associated with an increased risk for encephalocele (*OR* = 2.52, 95% *CI*, 1.25–5.07; *OR* = 1.80, 95% *CI*, 1.04–3.12; and *OR* = 1.96, 95% *CI*, 1.12–3.45), compared with those harboring the alleles T, C, and T, respectively [99]. Meanwhile, a case–control study on the polymorphisms of BER gene *LIG3* in 108 NTD pregnant women and 233 normal healthy pregnant women found that the TT genotype of rs1052536 in *LIG3* increased the risk of anencephaly (*OR* = 2.69, 95% *CI*, 1.18–6.10) and T allele carriers had significantly increased risk of cranial NTDs (*OR* = 1.56, 95% *CI*, 1.04–2.35) [100]. These results indicate that BER gene mutation might be a potential genetic risk factor for NTD occurrence.

### 4.3. DSBR and NTDs

DNA double-strand breaks (DSBs) are the most serious type among all forms of DNA damage and are repaired by homologous recombination (HR) and nonhomologous DNA end joining (NHEJ) [101,102]. HR begins with the binding of the MRN complex (MRE11, RAD50 and NBS1) to the broken DNA ends [103]. MRN interacts with CtIP, promoting end resection [104]. Bulk resection occurs via activation of the nucleases exonuclease 1 (EXO1), DNA replication ATP-dependent helicase (DNA2), and the Bloom syndrome helicase (BLM), generating single-stranded DNA (ssDNA), which is rapidly bound by RPA. The RAD51-ssDNA nucleofilament is formed by replacing RPA with RAD51 [104]. The RAD51-ssDNA filament carries out homology search, then invades a homologous DNA sequence and forms a displacement loop (D-loop) [104]. DNA polymerase can subsequently add nucleotides at the free 3′end to restore the sequence of DNA damage [105]. In NHEJ, the DNA ends in DSBs are recognized by the Ku70/Ku80 heterodimer, which then recruits and activates the catalytic subunit of the DNA dependent protein kinase (DNA-PK). The compatible ends are created by nucleases such as Artemis [106,107]. The DNA ligase IV and XRCC4 complex ligate the ends [108,109,110] (Figure 4).

The DSBR pathway plays potential roles in neurodevelopment and neuronal function [111]. DSBR is also influenced by folate status. Folate deficiency increases the expression of (phosphorylated H2AX) γ-H2AX, a DNA injury marker protein, as well as the methylation frequency of CpG site in the DSBR gene *Rad54* promoter region, and downregulates the expression of Rad54 in mouse sperm and spermatocyte line with folate deficiency [112]. The study of pathogenesis of NTDs has demonstrated that the E3 ligase mouse double minute 2 homolog (Mdm2) colocalized with γ-H2AX is recruited to the sites of DSBs in mouse embryonic stem cells (mESCs) after exposure to methotrexate (a folate antagonist [16]), which might contribute to NTDs induced by folate deficiency [113]. The DSBs in mESCs with folate deficiency were most prominently enriched in intergenic regions of the genome (45.7%), followed by the intronic and exonic regions (25.2% and 23.24%, respectively) [15]. These studies indicate that DSBs caused by folate deficiency is predisposition for NTDs. Furthermore, it was reported that the DSBR gene *XRCC3* (Thr241Met) polymorphism reduced the risk of spina bifida from a case–control study by the California birth defects monitoring program [10]. Inositol-5,6-kinase 1,3,4-triphosphate (ITPK1) is a key enzyme for inositol hexaphosphate (IP6) synthesis. Down-regulating the expression of *ITPK1* in transgenic mice through gene capture technology induced NTDs, bone defects, and growth retardation [114]. IP6 can activate NHEJ by binding to Ku protein in a DNA-PK complex and changing its conformation [115,116]. Therefore, the occurrence of NTDs caused by the down-regulation of ITPK1 might be caused by the reduction of IP6 synthesis, which eventually leads to the obstruction of the NHEJ process. Telomerase RNA knockout (*mTR^−/−^*) mice exhibited NTDs, which is related to telomere deletion, chromosomal instability, and apoptosis [117]. In *mTR^−/−^* mice, cells lacking Ku70/86 protein were more prone to apoptosis. Ku70/86, a member of the DNA-PK complex, is involved in NHEJ, which can repair dysfunctional telomeres [118]. It is speculated that loss of Ku protein will cause NHEJ disorder, resulting in abnormal apoptosis and eventually leading to NTDs. These studies suggest that both DSB induced by folate deficiency and DSBR gene mutation are related to NTDs.

Although DNA repair pathways (such as BER, MMR, DSBR) have been studied for many years, a complete understanding of how these pathways function in the pathogenesis of NTDs is still lacking. Given the intricate complexity of human NTDs, delineation of the relationship between folate and DNA repair pathways during neural tube closure remains an attractive field. Understanding how impairment of the regulatory mechanisms of DNA repair pathways may increase the risk of NTDs will help develop novel therapeutic approaches to reduce NTD occurrence in future. Here, the gene mutations in DNA repair pathways most likely lead to NTDs during the period of neural tube closure. Other malformations were also observed at the early formation of critical organ systems. The BER gene *Xrcc1* mutant embryos demonstrated increased cell death in the epiblast and an altered morphology in the visceral embryonic endoderm at embryonic day 6.5–7.5 [119]. The degeneration in *Apex1* gene homozygous mutant embryos were observed at embryonic day 5.5 [120]. These data suggest that mutations in DNA repair genes leads to dysfunction in certain tissues or organs and disturbed genomic integrity during the embryonic period, resulting in embryo developmental anomalies. Folate deficiency might cause mutations or abnormal methylation of the gene in DNA repair pathways, promote genomic instability, and alter gene expression. This might impair the balance of apoptosis and proliferation during the development of the neural tube. DNA repair pathways might be a link between folate deficiency and NTDs.

In recent years, there have been few studies focusing on the mechanism of the DNA repair pathway and NTDs with folate deficiency. Studies have shown that folate insufficiency promotes increase of rare variants through the loss of MMR, resulting in more severe MMR deficiency, providing a mechanistic link between low folate status and the occurrence of NTDs through deficits in MMR machinery [79]. It has been reported that biological behavior is similar between tumorigenesis and early embryonic development [121]. Some studies found that alteration of folate-metabolism-related enzymes affecting genomic stability was closely related to the function of DNA repair pathways in cancers, such as methylenetetrahydrofolate reductase (MTHFR) [122,123], prostate-specific membrane antigen (PSMA) [124], mitochondrial methylenetetrahydrofolate dehydrogenase (MTHFD2) [125] and Folylpolyglutamate synthetase (FPGS) [126]. Therefore, mutation in DNA repair pathways or folate-metabolism-related enzymes could cause genomic instability, impair the balance of cell proliferation and apoptosis, and, finally, lead to the occurrence of NTDs.

## 5. Conclusions

In summary, efficient DNA repair is essential to remove DNA lesions and aberrant structures and maintain genome integrity and stability for embryonic neural tube development. Folate deficiency not only blocks the synthesis of dTMP and promoted uracil misincorporation, but also causes DNA hypomethylation by reducing the production of methyl donors, which results in genomic instability, such as base mismatch, DNA breakage, and even chromosome aberration. Thus, it was speculated that, under the condition of folate deficiency, abnormal methylation modification or mutation in DNA repair genes are the main mechanisms of NTDs and folate deficiency. Abnormal DNA repair genes, which attenuate the capacity of DNA repair, cause abnormal gene expression, resulting in the imbalance of cell proliferation and apoptosis at the cellular level, which leads to incomplete neural tube closure and, ultimately, the occurrence of NTDs. Although maternal folate supplement is effective for ameliorating the risk of NTDs, the underlying mechanism of their action remains unclear. It is important to investigate how folate deficiency affects DNA repair pathways and how DNA repair pathways impact NTDs. A more complete understanding of the association between folate deficiency and DNA repair pathways during neural tube development would be insightful to support the mechanistic framework of nutrient-based therapeutics in reducing the incidence of embryonic developmental anomalies. The study of NTDs from the aspect of the DNA repair pathway will provide more comprehensive understanding of the pathogenesis of NTDs.

## Data Availability

Not applicable.
